# Novel H5N6 reassortants bearing the clade 2.3.4.4b HA gene of H5N8 virus have been detected in poultry and caused multiple human infections in China

**DOI:** 10.1080/22221751.2022.2063076

**Published:** 2022-04-25

**Authors:** Wenli Gu, Jianzhong Shi, Pengfei Cui, Cheng Yan, Yaping Zhang, Congcong Wang, Yuancheng Zhang, Xin Xing, Xianying Zeng, Liling Liu, Guobin Tian, Yasuo Suzuki, Chengjun Li, Guohua Deng, Hualan Chen

**Affiliations:** a State Key Laboratory of Veterinary Biotechnology, Harbin Veterinary Research Institute, CAAS, Harbin, People’s Republic of China; b Guangdong Laboratory for Lingnan Modern Agriculture, Guangzhou, People’s Republic of China; c Department of Medical Biochemistry, University of Shizuoka School of Pharmaceutical Sciences, Shizuoka, Japan

**Keywords:** Avian influenza virus, H5N6, clade2.3.4.4b, evolution, pathogenicity, receptor-binding properties

## Abstract

The globally circulating H5N8 avian influenza viruses bearing the clade 2.3.4.4b hemagglutinin (HA) gene are responsible for the loss of more than 33 million domestic poultry since January 2020. Moreover, the H5N8 viruses have reassorted with other avian influenza viruses and formed H5N1, H5N2, H5N3, H5N4, and H5N5 viruses in Europe, Africa, and North America. In this study, we analyzed 15 H5N6 viruses isolated from poultry and seven H5N6 viruses isolated from humans, and found these viruses formed seven different genotypes by deriving the clade 2.3.4.4b HA gene of H5N8 viruses, the neuraminidase of domestic duck H5N6 viruses, and internal genes of different viruses that previously circulated in domestic ducks and wild birds in China. Two of these genotypes (genotype 3 and genotype 6) have caused human infections in multiple provinces. The H5N6 viruses isolated from poultry have distinct pathotypes in mice; some of them replicate systemically and are highly lethal in mice. Although these viruses exclusively bind to avian-type receptors, it is worrisome that they may obtain key mutations that would increase their affinity for human-type receptors during replication in humans. Our study indicates that the novel H5N6 reassortants bearing the clade 2.3.4.4b HA gene of H5N8 viruses were generated through reassortment in domestic ducks and may have spread across a wide area of China, thereby posing a new challenge to the poultry industry and human health. Our findings emphasize the importance of careful monitoring, evaluation, and control of the H5N6 viruses circulating in nature.

## Introduction

Influenza A viruses are divided into different subtypes according to the antigenicity of their viral glycoproteins: hemagglutinin (HA) and neuraminidase (NA). Currently, 16 different HA (H1–H16) and nine different NA (N1–N9) subtypes have been identified from avian species, and the H17N10 and H18N11 subtypes have been detected in bats [1,2]. In recent years, the H5 and H7 subtypes of highly pathogenic viruses have caused avian influenza outbreaks in poultry and wild birds in many countries [3–10], leading to disastrous consequences for the poultry industry. In addition, these viruses have transmitted to humans and caused severe disease and deaths [11–13]. The HA of H5 viruses has evolved into different phylogenetic clades, from clade 0 to clade 9, and some of these clades have been further divided into subclades [14]. The clade 2.3.4.4 HA of H5 viruses has been further divided into eight subclades, namely clades 2.3.4.4a to 2.3.4.4h [15]. H5 viruses bearing the clade 2.3.4.4b HA gene are currently widely detected in poultry and wild birds in many countries in Africa, Europe, Asia, and Northern America [6–9,11], and have caused the loss of more than 33 million domestic poultry around the world [16].

In January 2020, H5N8 viruses bearing the clade 2.3.4.4b HA gene caused outbreaks in chickens in Poland and then started a new wave of outbreaks in poultry and wild birds globally [7,9,17]. Cui et al. performed a detail analysis of the spatiotemporal spread and genetic evolution of the H5N8 viruses, and found that the HA of H5N8 viruses formed two branches (branch I and branch II) that probably separated in early 2018. The viruses in branch I circulated in domestic poultry and wild birds in Poland, Hungary, Germany, and the Czech Republic in the spring and summer of 2020, and were subsequently detected in domestic poultry and wild birds in Japan and South Korea in the winter of 2020. In January 2021, they were detected in whopper swans in China. The H5N8 viruses bearing the branch II HA were first detected in chickens in Iraq in May 2020, then caused multiple disease outbreaks in domestic poultry in July and August 2020 in Russia, and were subsequently detected in many countries in the Middle East, Europe, Africa, and Asia. The H5N8 viruses carrying the branch II HA gene began to be detected in swans and other wild birds in China from October 2020, and were also detected in domestic ducks and geese in 2021 [8].

During our routine surveillance, in addition to isolating the H5N8 viruses we reported previously [8], we also isolated some H5N6 viruses bearing the HA of clade 2.3.4.4b between December 2020 and December 2021. According to publicly available information, 24 human cases were confirmed to be caused by H5N6 viruses bearing the clade 2.3.4.4b HA gene in 2020 and 2021 in China (Table S1). In this study, we performed a detailed analysis to understand the genetic relationship of the H5N6 viruses isolated from poultry and humans, their virulence, and receptor-binding properties. Our study provides important information on the emergence and evolution of the H5N6 viruses bearing the clade 2.3.4.4b HA gene, and evaluates its risk to humans.

## Materials and methods

### Ethics statements and facility

Swab samples collected during surveillance were processed in the enhanced biosafety level 2 (BSL2+) facility in the Harbin Veterinary Research Institute of the Chinese Academy of Agricultural Sciences (HVRI, CAAS). All experiments with live H5N6 viruses or organs collected from dead birds were carried out in the animal biosafety level 3 (ABSL3) facility in the HVRI. The study was carried out in strict accordance with the recommendations in the Guide for the Care and Use of Laboratory Animals of the Ministry of Science and Technology of China. The protocol for the animal studies was approved by the Committee on the Ethics of Animal Experiments of the HVRI, CAAS.

### Virus isolation

Swab samples or organs from dead birds were individual inoculated into 10-day-old embryonated chicken eggs and incubated for 48 h at 37°C. The HA subtype was identified by using the hemagglutinin inhibition (HI) test and the NA subtype was confirmed by direct sequence analysis. The H5N6 viruses were biologically cloned three times by limiting dilution in embryonated specific-pathogen-free (SPF) chicken eggs, and the virus stocks were grown in SPF chicken eggs and maintained at −70°C.

### Genetic and phylogenetic analyses

The genome of the H5N6 viruses was sequenced on an Applied Biosystems DNA Analyzer (3500xL Genetic Analyzer, USA). The nucleotide sequence was edited by using the Seqman module of the DNAStar package. The phylogenetic relationship of the HA gene of the H5 viruses was inferred with BEAST v1.8.4 by using a molecular clock that placed a timescale on virus evolution to study the recent evolutionary history of the novel H5N6 viruses in China. The HA gene of H5 representative viruses was downloaded from the Global Initiative on Sharing All Influenza Data databases (GISAID; https://www.gisaid.org) and National Center for Biotechnology Information (NCBI; https://www.ncbi.nlm.nih.gov). Phylogeny was estimated within a Bayesian Markov Chain Monte Carlo (MCMC) framework by using the GTR model and 50 million steps of MCMC, Bayesian skyline models of population dynamics and uncorrelated lognormal local clocks. Convergence was assessed with ESS (effective sample size) values after a burn-in of 5 million steps. Maximum-clade-credibility trees were generated using Tree Annotator from the BEAST package, and FigTree v1.4.4 was used to visualize the annotated trees. Phylogenetic analysis of the NA gene and the six internal genes was performed by using the MEGA 7.0 software package, implementing the neighbor-joining method. The tree topology was evaluated by 1,000 bootstrap analyses. The NA gene and six internal genes of representative influenza viruses were downloaded from the GISAID database.

### Receptor-binding analysis

The receptor specificity of the H5N6 viruses was determined by using a solid-phase direct binding test as previously described [18–20], with two glycopolymers: α-2, 3-sialylglycopolymer [Neu5Acα2-3Galb1-4GlcNAcb1-pAP (para-aminophenyl)-alpha-polyglutamic acid (α-PGA)] and α-2, 6-sialylglycopolymer [Neu5Acα2-6Galb1-4GlcNAcb1-pAP (para-aminophenyl)-alpha-polyglutamic acid (α-PGA)]. In this study, we employed chicken antiserum generated by a DNA vaccine encoding the HA gene of the H5N8 virus A/whooper swan/Shanxi/4-1/2020, as well as a horseradish peroxidase (HRP)-conjugated goat-anti-chicken antibody (Sigma-Aldrich, St. Louis, MO, USA).

### Replication and virulence of H5N6 viruses in mice

Groups of three 6-week-old female BALB/c mice (Beijing Experimental Animal Center, Beijing, China) were lightly anesthetized with CO_2_ and then inoculated intranasally (i.n.) with 10^6^ EID_50_ of H5N6 virus in a volume of 50 μl, and their organs, including nasal turbinate, lungs, spleen, kidneys, and brain were collected on Day 3 post-inoculation (p.i.) for virus titration in chicken eggs. To assess the 50% mouse lethal dose (MLD_50_), groups of five mice were inoculated with 10-fold serial dilutions of the test virus containing 10^1^–10^6^ EID_50_, and body weight loss and mortality were monitored for 14 days.

### Antigenic analysis

Antigenic analysis of the viruses was performed by using the HI assay with 1.0% chicken erythrocytes. The chicken antisera used in this assay were generated in 6-week-old SPF chickens. Briefly, chickens were inoculated with 0.5 mL of oil-emulsified inactivated vaccine seed virus or vaccine, and serum was collected three weeks after vaccination. H5-Re11, H5-Re12, H5-Re13, and H5-Re14 are vaccine seed viruses generated by reverse genetics; their surface genes are derived from A/duck/Guizhou/S4184/2017(H5N6) (a clade 2.3.4.4h virus), A/chicken/Liaoning/SD007/2017(H5N1) (a clade 2.3.2.1d virus), A/duck/Fujian/S1424/2020(H5N6) (a clade 2.3.4.4h virus), and A/whooper swan/Shanxi/4-1/2020(H5N8) (a clade 2.3.4.4b virus), respectively, and their internal genes from the A/Puerto Rico/8/1934 (H1N1) (PR8) virus. The H5 bivalent vaccine (H5-Re13 + H5-Re14) and H5/H7 trivalent vaccine (H5-Re13 + H5-Re14 + H7-Re4) are the updated vaccines currently used in poultry in China. The H7-Re4 seed virus was generated by reverse genetics; its surface genes are derived from A/chicken/Yunnan/SD024/2021(H7N9), which was isolated from a chicken during our routine surveillance [21].

## Results

### Virus isolation and identification

From the samples we collected between December 2020 and December 2021, we isolated 15 H5N6 viruses that bear the HA gene of the clade 2.3.4.4b (Table 1): 11 H5N6 viruses were isolated from domestic duck and goose swabs collected in the live poultry markets, and four viruses were isolated from the lung samples of two dead chickens, a dead domestic duck, and a dead goose that were raised in free-range households (Table 1). These viruses were isolated from samples collected from Guangdong, Sichuan, Chongqing, Shaanxi, Guangxi, Yunnan, Guizhou, Hunan, and Zhejiang provinces.

**Table 1.** H5N6 viruses bearing the clade 2.3.4.4b HA gene isolated in this study.

Virus			Sample information		
Full name	Abbreviation		Sample type	Collected date	Location
A/duck/Guangdong/S4879/2020(H5N6)	DK/GD/S4879/2020		Swab	December 3, 2020	Poultry market
A/duck/Guangdong/SE195/2021(H5N6)	DK/GD/SE195/2021		Swab	January 11, 2021	Poultry market
A/duck/Sichuan/S1805/2021(H5N6)	DK/SC/S1805/2021		Swab	April 8, 2021	Poultry market
A/chicken/Shaanxi/SD001/2021(H5N6)	CK/SN/SD001/2021		Lung	May 21, 2021	Free-range household
A/chicken/Chongqing/SD001/2021(H5N6)	CK/CQ/SD001/2021		Lung	July 13, 2021	Free-range household*
A/duck/Sichuan/SD002/2021(H5N6)	DK/SC/SD002/2021		Lung	July 26, 2021	Free-range household
A/goose/Chongqing/SD003/2021(H5N6)	GS/CQ/SD003/2021		Lung	September 25, 2021	Free-range household*
A/duck/Guangxi/S30428/2021(H5N6)	DK/GX/S30428/2021		Swab	October 18, 2021	Poultry market
A/duck/Guangxi/S31116/2021(H5N6)	DK/GX/S31116/2021		Swab	October 18, 2021	Poultry market
A/duck/Yunnan/S4318/2021(H5N6)	DK/YN/S4318/2021		Swab	November 2, 2021	Poultry market
A/duck/Hunan/S40199/2021(H5N6)	DK/HuN/S40199/2021		Swab	December 1, 2021	Poultry market
A/duck/Hunan/S40268/2021(H5N6)	DK/HuN/S40268/2021		Swab	December 1, 2021	Poultry market
A/duck/Guizhou/S4702/2021(H5N6)	DK/GZ/S4702/2021		Swab	December 2, 2021	Poultry market
A/duck/Zhejiang/S4854/2021(H5N6)	DK/ZJ/S4854/2021		Swab	December 3, 2021	Poultry market
A/goose/Guangdong/S4751/2021(H5N6)	GS/GD/S4751/2021		Swab	December 8, 2021	Poultry market

*Human case was also detected in the house.

### Phylogenic analysis of H5N6 viruses bearing the clade 2.3.4.4b HA gene isolated from poultry and humans in China

**Figure 1.**
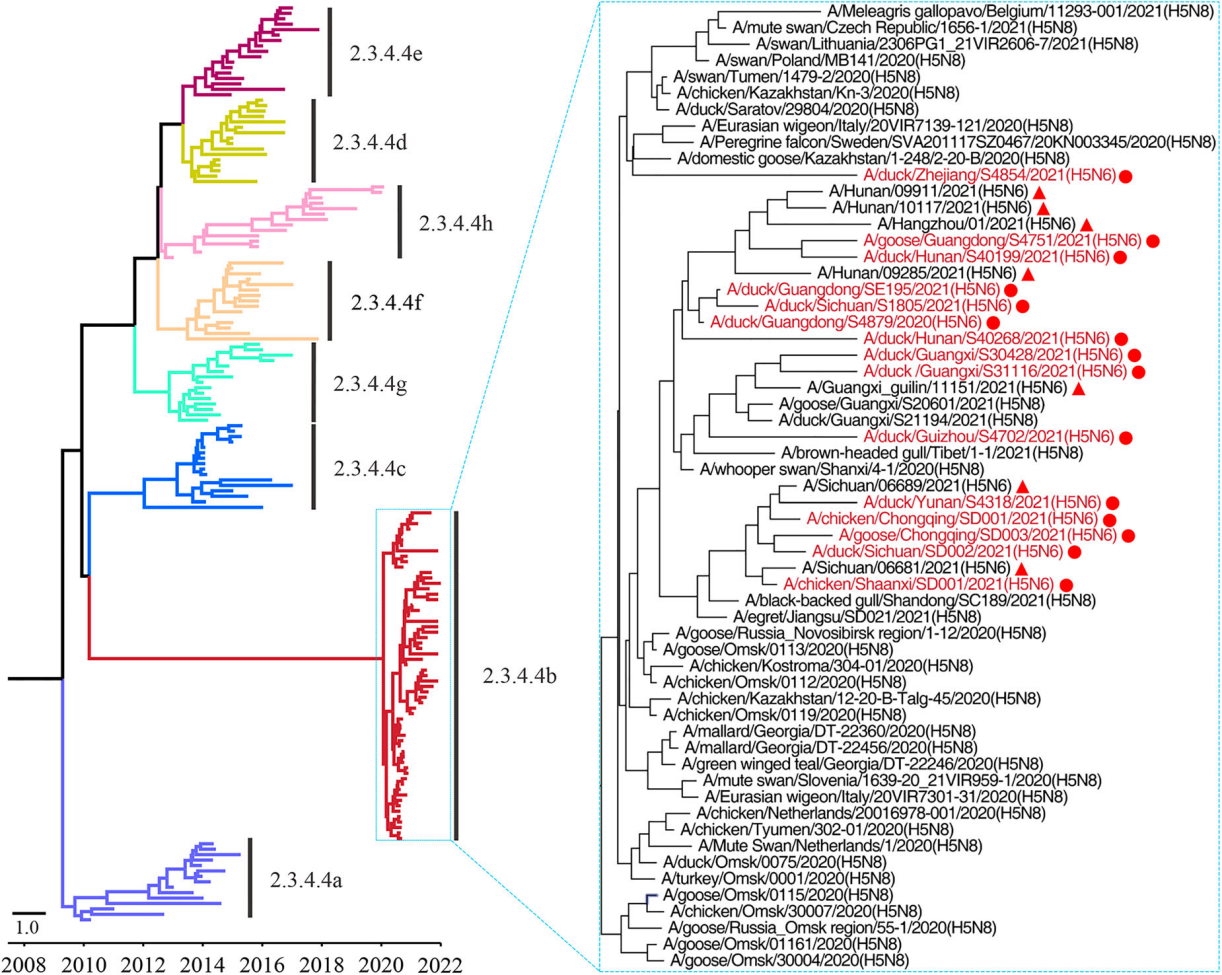
Phylogenetic tree of the HA genes of H5 viruses. The Bayesian time-resolved phylogenetic tree of the clade 2.3.4.4 HA gene of 170 H5 viruses, including the 15 novel H5N6 reassortants in this study and 155 representative viruses reported by others. The phylogenetic tree of the HA gene with more complete information is shown in the Supporting Figure S1 and was rooted to A/goose/Guangdong/1/1996 (H5N1). The viral names of the 59 clade 2.3.4.4b HAs are shown in the small tree, in which the novel H5N6 viruses sequenced in this study are shown in red and marked with red dots. The H5N6 viruses isolated from humans are marked with red solid triangles.

To investigate the genetic relationship of these strains, we fully sequenced the whole genome of the 15 H5N6 viruses (the sequence data have been deposited in GISAID under accession numbers EPI1981575–EPI1981582, EPI1981593–EPI1981640, and EPI1997171–EPI1997234), and performed a phylogenic analysis with representative strains downloaded from public databases, including the H5N8 viruses detected around the world since 2020 and the seven human H5N6 viruses isolated from patients in the Sichuan, Guangxi, Hunan, and Zhejiang provinces of China that have been previously deposited by others (Figure 1). We found that the HA genes of the 15 viruses shared 97.7%–100% identity at the nucleotide level with the HA gene of other viruses in the clade 2.3.4.4b (Figure 1). The HA gene of the H5N6 viruses isolated from poultry and humans and the H5N8 viruses detected since 2020 clustered together in the Bayesian time-resolved phylogenetic tree that was generated with the HA gene of representative H5 viruses of different clades (clade 2.3.4.4a to clade 2.3.4.4h) (Figure 1, Figure S1).

**Figure 2.**
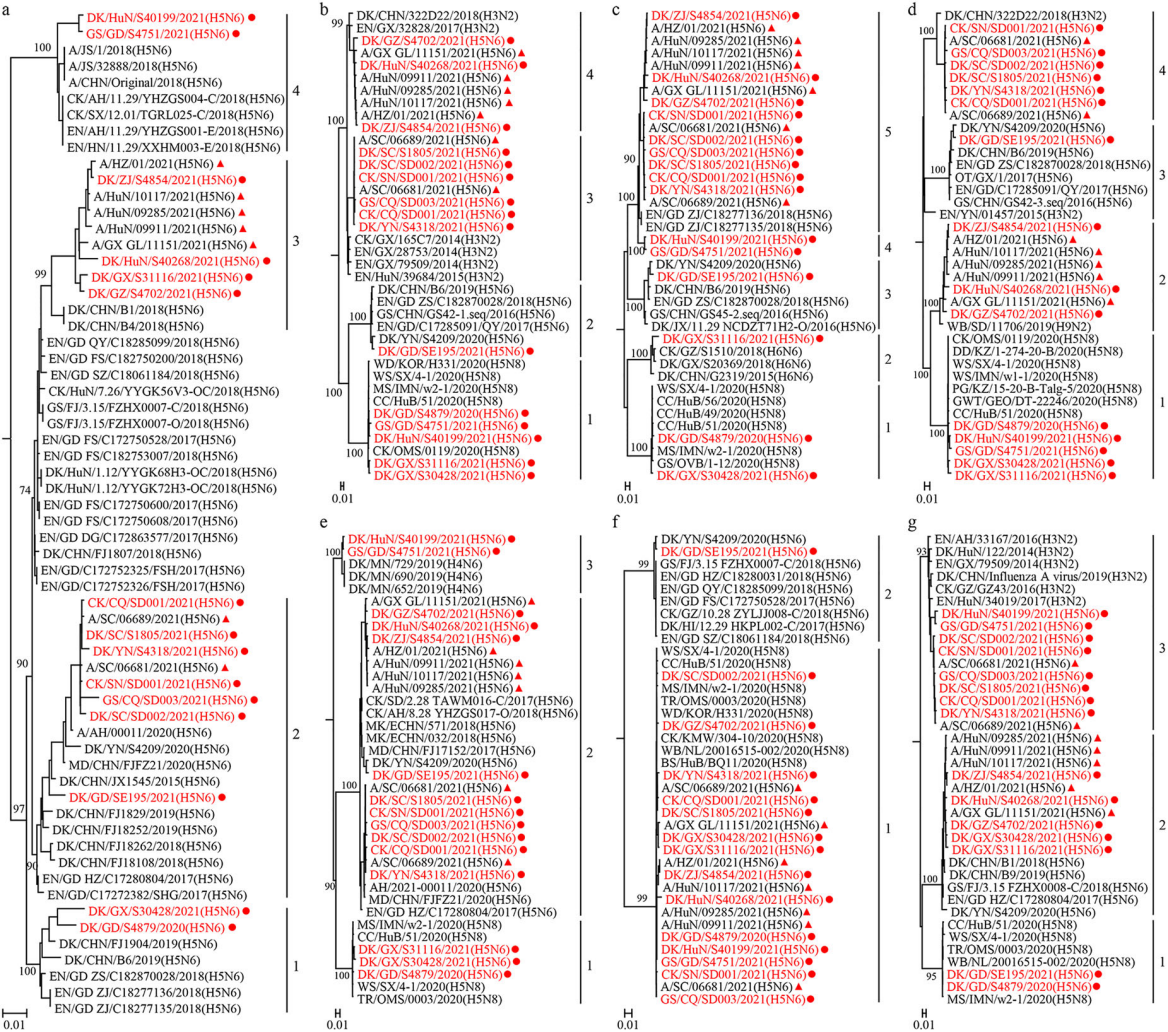
Phylogenetic analyses of the NA gene and the six internal genes of the H5N6 viruses. Phylogenetic analysis was performed by using the MEGA 7.0 software package, implementing the neighbor-joining method. The tree topology was evaluated by 1,000 bootstrap analyses. (a) The phylogenetic tree of the NA genes of 63 H5N6 viruses was rooted to A/Changsha/1/2014 (H5N6). (b-g) The phylogenetic trees of the PB2, PB1, PA, NP, M, and NS genes were rooted to A/goose/Guangdong/1/1996 (H5N1). The novel H5N6 viruses sequenced in this study are shown in red and marked with red dots. The H5N6 viruses isolated from humans are marked with red solid triangles.

The NA genes of the 15 H5N6 viruses shared 93.2%–99.9% identity at the nucleotide level and formed four groups in the phylogenetic tree; they were closely related to the NA gene of the H5N6 viruses previously detected in domestic ducks (Figure 2(a)). The six internal genes of the novel H5N6 viruses showed distinct diversity, with the basic polymerase 2 (PB2), basic polymerase 1 (PB1), acidic polymerase (PA), nucleoprotein (NP), matrix (M), and nonstructural protein (NS) genes of the 15 viruses sharing 84.9%–99.9%, 88.1%–99.9%, 89.9%–99.9%, 89.3%–100%, 91%–100%, and 86.7%–100% identity, respectively, at the nucleotide level. The PB2 and PA genes each formed four groups in the phylogenetic trees (Figure 2(b, d)); the PB1 gene of these viruses formed five groups in the phylogenetic tree (Figure 2(c)); the NP and NS genes each formed three groups in their phylogenetic trees (Figure 2(e, g)); and the M gene of these viruses formed two groups in the phylogenetic tree (Figure 2(f)). Of note, the gene segments of seven human viruses clustered with the poultry H5N6 viruses detected in this study (Figure 2). These results indicate that the NA and internal genes of the H5N6 viruses isolated from poultry and humans in China show clear diversity.

### Genotypic analysis of the H5N6 viruses detected in poultry and humans in China

**Figure 3.**
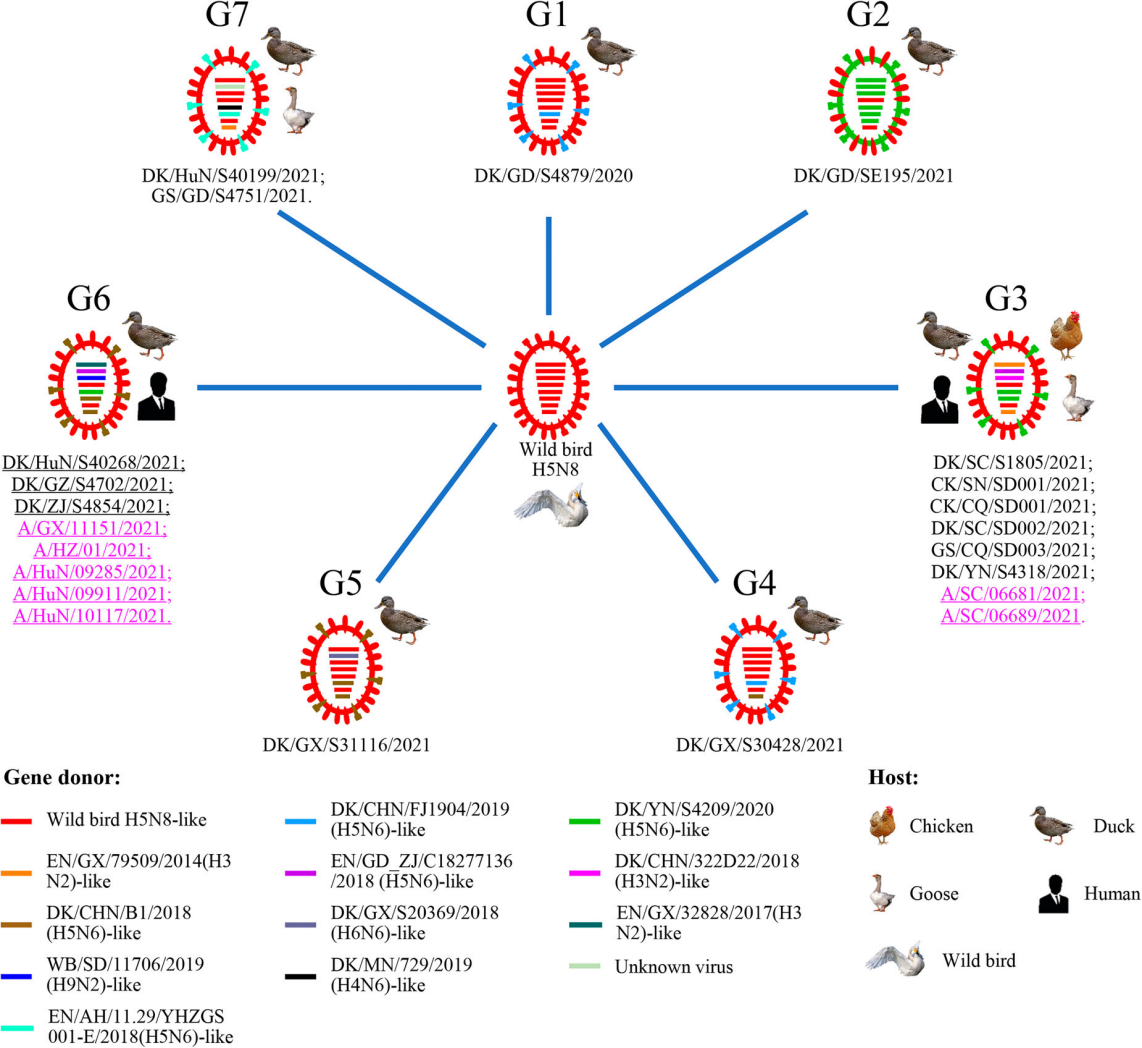
Genotypes of the H5N6 viruses and the hosts from which these genotypes were detected. The eight bars represent the eight gene segments (from top to bottom: PB2, PB1, PA, HA, NP, NA, M, and NS), and the colour of the bar indicates the closest donor strain of the gene segment.

On the basis of their genomic differences, the 15 H5N6 viruses isolated from poultry and the seven H5N6 viruses isolated from humans formed seven different genotypes (G1 to G7), which resulted from frequent reassortment with other wild bird and domestic duck viruses (Figure 3). The G1 virus, DK/GD/S4879/2020, derived its seven genes from an H5N8 wild bird virus and its NA gene from an H5N6 domestic duck virus; the G2 virus, DK/GD/SE195/2021, derived its HA and NS genes from an H5N8 wild bird virus and the other six genes from an H5N6 domestic duck virus; the G3 viruses, DK/SC/S1805/2021, CK/SN/SD001/2021, CK/CQ/SD001/2021, DK/SC/SD002/2021, GS/CQ/SD003/2021, DK/YN/S4318/2021, A/SC/06681/2021, and A/SC/06689/2021, derived their HA and M genes from an H5N8 wild bird virus, their PB2, PA, and NS genes from different H3N2 domestic duck viruses, and their PB1, NP and NA from different H5N6 domestic duck viruses; the G4 virus, DK/GX/S30428/2021, derived its six genes from an H5N8 wild bird virus, and its NA and NS genes from different H5N6 domestic duck viruses; the G5 virus, DK/GX/S31116/2021, derived its five genes from an H5N8 wild bird virus, its NA and NS genes from an H5N6 domestic duck virus, and its PB1 gene from an H6N6 domestic duck virus; the G6 viruses, DK/HuN/S40268/2021, DK/GZ/S4702/2021, DK/ZJ/S4854/2021, A/GX/11151/2021, A/HZ/01/2021, A/HuN/09285/2021, A/HuN/09911/2021, and A/HuN/10117/2021, derived their HA and M genes from an H5N8 wild bird virus, their PB2 and PA from an H3N2 domestic duck virus and an H9N2 wild bird virus, respectively, and their PB1, NP, NA, and NS from three different H5N6 viruses; and the G7 viruses, DK/HuN/S40199/2021 and GS/GD/S4751/2021, derived their four genes from an H5N8 wild bird virus, their PB1 genes from unknown virus, their NA from an H5N6 domestic duck virus, their NP gene and NS gene from an H4N6 domestic duck virus and an H3N2 domestic duck virus, respectively (Figure 3). The G1, G2, G4, and G5 viruses were only detected in domestic ducks, the G7 viruses were detected in a domestic duck and a goose, the G3 viruses were detected in domestic ducks, geese, chickens, and humans, and the G6 viruses were detected in domestic ducks and humans. These analyses indicate that the wild bird H5N8 viruses have undergone complicated reassortments since they were introduced into domestic ducks in China, and some of the reassortants have spread across wide geographic areas and infected different avian species and humans.

### Molecular analysis of the H5N6 viruses

The novel H5N6 viruses have the same polybasic amino acid motif of -RRKR/GLF- in their HA cleavage site, which is a characteristic of highly pathogenic avian influenza virus. Although the Q226L or G228S amino acid substitutions were not detected in the HA, all 22 viruses have the amino acids 137A, 158N, 160A, and 186N in their HA, and 20 viruses have the amino acid 192I in their HA (H3 numbering), which have been reported to increase the affinity of H5 influenza virus for human-type receptors (Table 2). The E627K or D701N substitutions in PB2 that increase virulence in mammals were not detected in these H5N6 viruses; however, the following amino acid residues linked to increased replication and virulence in mammals were detected in these H5N6 viruses: 225G in HA (H3 numbering) [22], 89V, 292V (six of 22 viruses), 309D, 389R, and 598T in PB2, 622G in PB1, 30D, 43M, and 215A in M1, and 42S and 106M in NS1 (Table 2).

**Table 2.** Mutations detected in the H5N6 viruses that contribute to the increased binding to human-type receptors and virulence in mammals.

Virus	Genotype	Amino acids in HA that increase affinity to human-type receptors (H3 numbering)		Amino acids that increase the replication and virulence of avian influenza viruses in mammals					
				HA	PB2		PB1	M1	NS1
		137/158/160/186	192	225	89/309/389 /598	292	622	30/43/215	42/106
DK/GD/S4879/2020	G1	A/N/A/N	I	G	V/D/R/T	V	G	D/M/A	S/M
DK/GD/SE195/2021	G2	A/N/A/N	I	G	V/D/R/T	V	G	D/M/A	S/M
DK/SC/S1805/2021	G3	A/N/A/N	I	G	V/D/R/T	/^b^	G	D/M/A	S/M
CK/SN/SD001/2021	G3	A/N/A/N	I	G	V/D/R/T	/	G	D/M/A	S/M
CK/CQ/SD001/2021	G3	A/N/A/N	I	G	V/D/R/T	/	G	D/M/A	S/M
DK/SC/SD002/2021	G3	A/N/A/N	I	G	V/D/R/T	/	G	D/M/A	S/M
GS/CQ/SD003/2021	G3	A/N/A/N	I	G	V/D/R/T	/	G	D/M/A	S/M
DK/YN/S4318/2021	G3	A/N/A/N	I	G	V/D/R/T	/	G	D/M/A	S/M
A/SC/06681/2021^a^	G3	A/N/A/N	I	G	V/D/R/T	/	G	D/M/A	S/M
A/SC/06689/2021^a^	G3	A/N/A/N	I	G	V/D/R/T	/	G	D/M/A	S/M
DK/GX/S30428/2021	G4	A/N/A/N	I	G	V/D/R/T	V	G	D/M/A	S/M
DK/GX/S31116/2021	G5	A/N/A/N	I	G	V/D/R/T	V	G	D/M/A	S/M
DK/HuN/S40268/2021	G6	A/N/A/N	I	G	V/D/R/T	/	G	D/M/A	S/M
DK/GZ/S4702/2021	G6	A/N/A/N	I	G	V/D/R/T	/	G	D/M/A	S/M
DK/ZJ/S4854/2021	G6	A/N/A/N	/	G	V/D/R/T	/	G	D/M/A	S/M
A/GX/11151/2021^a^	G6	A/N/A/N	I	G	V/D/R/T	/	G	D/M/A	S/M
A/HZ/01/2021^a^	G6	A/N/A/N	I	G	V/D/R/T	/	G	D/M/A	S/M
A/HuN/09285/2021^a^	G6	A/N/A/N	I	G	V/D/R/T	/	G	D/M/A	S/M
A/HuN/09911/202^a^	G6	A/N/A/N	I	G	V/D/R/T	/	G	D/M/A	S/M
A/HuN/10117/2021^a^	G6	A/N/A/N	I	G	V/D/R/T	/	G	D/M/A	S/M
DK/HuN/S40199/2021	G7	A/N/A/N	/	G	V/D/R/T	V	G	D/M/A	S/M
GS/GD/S4751/2021	G7	A/N/A/N	I	G	V/D/R/T	V	G	D/M/A	S/M

^a^ Sequences of human H5N6 viruses were downloaded from the GISAID.

^b^ No such mutation.

### Receptor-binding properties of the H5N6 reassortants

**Figure 4.**
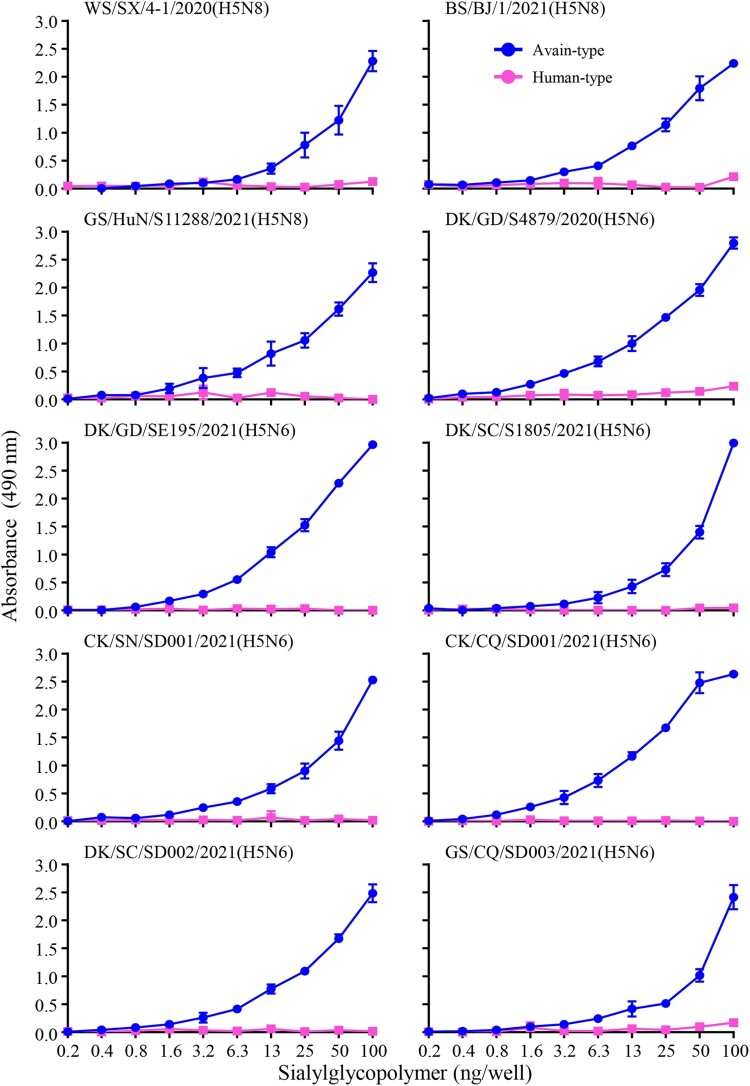
Receptor-binding properties of the H5N6 and H5N8 viruses bearing the clade 2.3.4.4b HA gene. Binding of the indicated viruses to sialylglycopolymers (α-2,3-sialylglycopolymer, blue; α-2,6-sialylglycopolymer, pink). The data shown are the means of three repeats; the error bars indicate standard deviations.

Binding to α-2,6-linked sialic acids (SAs) (human-type receptor) is a prerequisite for influenza virus to transmit efficiently among humans [23]. Since the H5N8 and H5N6 viruses bearing the clade 2.3.4.4b HA gene have caused human infections in Russia and China, respectively [24–26], we investigated their receptor-binding properties by evaluating the binding capacity to α-2,3-sialylglycopolymer (avian-type receptor) and α-2,6-sialylglycopolymer (human-type receptor) of ten viruses, including three H5N8 viruses that were reported previously [8] and the seven H5N6 viruses isolated in this study, by using solid-phase binding assays as described previously [18]. We found that all ten viruses exclusively bound to α-2,3-sialylglycopolymer (Figure 4). These results indicate that the H5N6 and H5N8 viruses bearing the clade 2.3.4.4b HA gene have retained their ability to bind to avian-type receptors and have not yet acquired the ability to bind to human-type receptors.

### Replication and virulence of H5N6 viruses in mice

**Figure 5.**
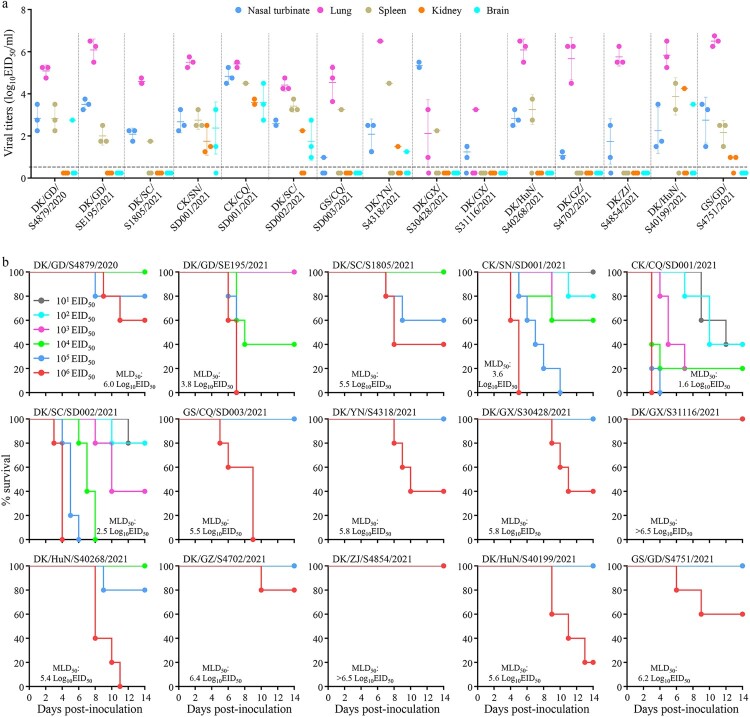
Replication and virulence of H5N6 viruses in mice. (a) Virus titers in organs of mice inoculated intranasally with 10^6^ EID_50_ of different H5N6 viruses. Three mice from each group were euthanized and their organs were collected on day 3 post-inoculation for virus titration in eggs. Data shown are means ± standard deviations. The dashed lines indicate the lower limit of detection. (b) The death pattern and MLD_50_ of the indicated viruses.

Mice have been widely used as model animals for evaluating the virulence of avian influenza viruses in mammals, and we therefore evaluated the replication and virulence of the 15 H5N6 viruses in mice as previously described [27–29]. We found that five viruses, CK/SN/SD001/2021, CK/CQ/SD001/2021, DK/SC/SD002/2021, DK/YN/S4318/2021, and DK/HuN/S40199/2021 replicated systemically and could be detected in all five mouse organs tested (Figure 5(a)); their 50% mouse lethal doses (MLD_50_) were 3.6 log_10_ 50% egg infectious doses (EID_50_), 1.6 log_10_ EID_50_, 2.5 log_10_ EID_50_, 5.8 log_10_ EID_50_, and 5.6 log_10_ EID_50_, respectively (Figure 5(b)). DK/GD/S4879/2020 was detected in the nasal turbinate, lungs, spleen, and brain of mice, but was not detected in the kidneys of any mice (Figure 5(a)); one mouse in the 10^5^ EID_50_-inoculated group and two mice in the 10^6^ EID_50_-inoculated group died during the observation period, yielding an MLD_50_ of 6.0 log_10_ EID_50_ (Figure 5(b)). GS/GD/S4751/2021 was detected in the nasal turbinate, lungs, spleen, and kidneys of mice, but was not detected in the brain of any mice (Figure 5(a)); only two mice in the 10^6^ EID_50_-inoculated group died, and yielding an MLD50 of 6.2 log_10_ EID_50_ (Figure 5(b)). Five H5N6 viruses, DK/GD/SE195/2021, DK/SC/S1805/2021, GS/CQ/SD003/2021, DK/GX/S30428/2021, and DK/HuN/S40268/2021, were detected in the nasal turbinates, lungs, and spleen of mice, but were not detected in the brain and kidneys of any mice (Figure 5(a)); and their MLD_50_ values were 3.8 log_10_ EID_50_, 5.5 log_10_ EID_50_, 5.5 log_10_ EID_50_, 5.8 log_10_ EID_50_, and 5.4 log_10_ EID_50_, respectively (Figure 5(b)). Three viruses, DK/GX/S31116/2021, DK/GZ/S4702/2021, and DK/ZJ/S4854/2021, were only detected in the nasal turbinates and lungs of mice (Figure 5(a)); and their MLD_50_ values were >6.5 log_10_ EID_50_, 6.4 log_10_ EID_50_, and >6.5 log_10_ EID_50_, respectively (Figure 5(b)). These results indicate that H5N6 viruses circulating in nature have distinct pathotypes in mice, and some strains replicate systemically and are highly lethal in mice.

### Cross-reactivity of the H5N6 reassortants with antisera induced by different vaccine seed viruses

Vaccination is the major strategy for the control of highly pathogenic avian influenza in China, and we have developed different vaccine strains to prevent the viruses in different clades or subclades [30–32]. The previously used vaccine was an H5/H7 trivalent inactivated vaccine produced with the H5-Re11, H5-Re12, and H7-Re3 vaccine seed strains (the donor of the surface genes of H7-Re3 is A/chicken/Inner Mongolia/SD010/2019 (H7N9)) [33,34], and the recently updated vaccines are the H5 bivalent vaccine (H5-Re13 + H5-Re14) and H5/H7 trivalent vaccine (H5-Re13 + H5-Re14 + H7-Re4). To investigate the cross-reactivity of antisera induced by different vaccine seed viruses, we tested the hemagglutinin inhibition (HI) antibody titers of the antisera against the 15 H5N6 viruses. We found that the HI antibody titer of H5-Re11, H5-Re12, H5-Re13, and H5-Re14 antisera against the homologous viruses was 512, whereas the HI titers of the H5-Re11 antiserum against the 15 H5N6 viruses were 4–32, which was 16- to 128-fold lower than that to the homologous titer the HI titers of the H5-Re12 antiserum against the 15 H5N6 viruses were 2–16, which was 32- to 256-fold lower than that to the homologous titer; the HI titers of the H5-Re13 antiserum against the 15 H5N6 viruses were 32–128, which was 4- to 16-fold lower than that to the homologous titer; the HI titers of the H5-Re14 antiserum against the H5N6 viruses were 256–1024, which was only 2-fold lower or higher than that to the homologous titer (Table 3). Similar results were also observed with antisera induced by the H5 bivalent and H5/H7 trivalent vaccines that are currently in use in China (Table 3). These results indicate that the antisera induced by different antigens reacted differently with the H5N6 viruses, and that the antisera induced by H5-Re14 or vaccines bearing the H5-Re14 seed virus cross-reacted well with the H5N6 viruses.

**Table 3.** Cross-reactive HI antibody titers of the novel H5N6 viruses with antisera induced by different vaccine seed viruses or vaccines.

Virus	Clade	HI antibody titer of antiserum induced by different antigens^a^					
		H5-Re11	H5-Re12	H5-Re13	H5-Re14	H5 bivalent inactivated vaccine (H5-Re13 + H5-Re14)	H5/H7 trivalent inactivated vaccine (H5-Re13 + H5-Re14 + H7-Re4)
H5-Re11	2.3.4.4h	**512**	32	512	128	512	512
H5-Re12	2.3.2.1d	32	**512**	64	32	64	64
H5-Re13	2.3.4.4h	32	4	**512**	4	**512**	**512**
H5-Re14	2.3.4.4b	16	4	32	**512**	**512**	**512**
DK/GD/S4879/2020	2.3.4.4b	8	2	32	256	256	512
DK/GD/SE195/2021	2.3.4.4b	32	16	128	1024	1024	2048
DK/SC/S1805/2021	2.3.4.4b	4	2	64	256	256	512
CK/SN/SD001/2021	2.3.4.4b	8	2	64	256	256	512
CK/CQ/SD001/2021	2.3.4.4b	4	2	64	256	256	512
DK/SC/SD002/2021	2.3.4.4b	4	2	64	256	256	512
GS/CQ/SD003/2021	2.3.4.4b	4	2	64	256	256	512
DK/GX/S30428/2021	2.3.4.4b	8	2	64	256	256	512
DK/GX/S31116/2021	2.3.4.4b	4	2	64	256	256	512
DK/YN/S4318/2021	2.3.4.4b	8	2	64	256	256	256
DK/HuN/S40199/2021	2.3.4.4b	8	2	64	256	256	512
DK/HuN/S40268/2021	2.3.4.4b	8	2	64	256	256	512
DK/GZ/S4702/2021	2.3.4.4b	4	2	64	256	256	512
DK/ZJ/S4854/2021	2.3.4.4b	8	2	64	256	256	512
GS/GD/S4751/2021	2.3.4.4b	8	2	64	256	256	512

^a^ Antisera were generated by vaccinating specific-pathogen-free chickens with the indicated oil-emulsified inactivated viruses or vaccines; the homologous titers are shown in bold.

## Discussion

The H5N8 influenza viruses bearing the clade 2.3.4.4b HA gene have been circulating in wild birds and domestic poultry in many countries since 2020. In the meantime, novel reassortants bearing the H5N8 HA and NA from other influenza viruses have been detected in nature. H5N1 viruses were detected in wild birds and domestic poultry in Russia and many countries in Europe, Africa, Asia, and North America [35–37]; H5N2 viruses were detected in wild birds in Serbia and domestic poultry in Taibei, China and Bulgaria [37,38]; H5N3 viruses were detected in wild birds in Denmark, France, Germany, Ireland, and Netherlands [37]; H5N4 were detected in wild birds in Germany, Netherlands, and Sweden [37]; and H5N5 viruses were detected in wild birds and domestic poultry in Iran and many countries in Europe [37,39,40]. In this study, we found that H5N8 wild bird viruses have encountered complex reassortment with local H5N6, H3N2, H4N6, and H6N6 domestic duck viruses, and with wild bird H9N2 virus, thereby producing multiple genotypes of H5N6 viruses. These findings indicate that the clade 2.3.4.4b HA gene has high compatibility with different NA genes, and given that the H5N8 viruses have been detected in a wide range of wild birds over a wide geographic area, it is highly likely that H5N7 and H5N9 viruses bearing the clade 2.3.4.4b HA will also be generated in nature. Studies have shown that gene constellation can alter the virulence and transmissibility of influenza virus [41,42], and therefore it will be important to monitor and carefully evaluate the biological properties of different subtypes of H5 viruses bearing the clade 2.3.4.4b HA.

The 15 H5N6 viruses we reported in this study were detected between December 2020 and December 2021 and formed seven different genotypes, indicating that the H5N6 viruses bearing the clade 2.3.4.4b HA gene have actively reassorted with the viruses maintained in local poultry. As shown in Table S1, according to the WHO and China CDC website, there have been 24 human cases of confirmed infection with H5N6 viruses bearing the clade 2.3.4.4b HA. The available sequences of the seven human viruses, which were isolated between June 2021 and December 2021, indicate that two different genotypes had jumped to humans. We do not know if the other five genotypes are also responsible for human infections, as the sequence of the virus responsible for the other 17 human cases is unknown.

Binding to human-type receptors is important for transmission of influenza virus from human to human. Previous studies have shown that only a few amino acid changes in HA can alter receptor-binding preference and make avian influenza viruses bind to human-type receptors with high affinity [18,43–45]; some viruses have been shown obtain such mutations after a single round of replication in mammals [18,46]. Since H5N8 and H5N6 viruses bearing the clade 2.3.4.4b HA can infect and replicate efficiently in humans and other mammals [8,24,26], they may acquire the key mutations in HA that promote binding to human-type receptors during their replication in mammals. The receptor-binding assay in our study indicated that both the H5N8 and H5N6 viruses isolated from avian species exclusively bind to avian-type receptors; nevertheless, it is important to monitor and carefully evaluate the receptor-binding properties of the H5N8 and H5N6 viruses isolated from humans.

In China, domestic ducks are reared often in open fields with no biosecurity measures, poorly vaccinated, and are mainly traded through the live poultry market system [47]. It is also common for people to purchase birds from live poultry markets and keep them in backyards. Therefore, influenza viruses carried by domestic ducks can be easily spread widely and transmitted to humans. Our previous study indicated that routinely vaccinated chickens and domestic ducks on poultry farms could be completely protected against wild bird H5N8 virus challenge, although there was a clear antigenic difference between the H5N8 viruses and the H5 vaccine strains previously used in China [8]. Detection of H5N8 viruses in domestic ducks and geese in a previous study [8] and the novel H5N6 reassortants in birds in live poultry markets and backyards in this study provide direct evidence for how novel influenza viruses are introduced and evolve in poultry in China, and highlight the urgent need to increase vaccination coverage of waterfowl and backyard poultry in China.

In summary, here we found that the H5N6 viruses bearing the clade 2.3.4.4b HA gene detected in poultry in 2020 and 2021 in China are novel reassortants of the globally spread H5N8 viruses and other viruses circulating in local ducks and wild birds. Importantly, some strains are highly lethal in mice and have caused severe disease and death in humans. Our study provides important information about the ecology and risk to human health of the H5N6 viruses bearing the clade 2.3.4.4b HA.

## Supplementary Material

Supplemental Material

Supplemental Material
