# Supplementary material for: Novel H5N6 reassortants bearing the clade 2.3.4.4b HA gene of H5N8 virus have been detected in poultry and caused multiple human infections in China
Source: Emerg Microbes Infect. 2022 Apr 25;11(1):1174–85. doi: 10.1080/22221751.2022.2063076 (PMC9126593; doi:10.1080/22221751.2022.2063076)
Supplement: Supplemental Material [file TEMI_A_2063076_SM3736.docx]

**Table S1. Information of the 24 confirmed human cases infected by H5N6 viruses bearing the clade 2.3.4.4b HA gene^a^.**

| Case | Province | Onset date | Gender | Age (year) | Hospitalization date | Condition at the time of reporting |
| --- | --- | --- | --- | --- | --- | --- |
| 1 | Anhui | 12/22/ 2020 | Male | 1 | 12/22/2020 | Fatal |
| 2 | Chongqing | 07/09/2021 | Male | 66 | 06/23/2021 | Critical |
| 3 | Chongqing | 09/16/2021 | Male | 72 | 09/19/2021 | Fatal |
| 4 | Guangdong | 07/31/2021 | Female | 52 | 07/31/2021 | Critical |
| 5 | Guangdong | 09/13/2021 | Male | 53 | 09/18/2021 | Severe |
| 6 | Guangdong | 10/20/2021 | Male | 52 | 10/21/2021 | Critical |
| 7^b^ | Guangxi | 07/06/2021 | Female | 61 | 07/09/2021 | Mild |
| 8 | Guangxi | 08/14/2021 | Female | 26 | 08/19/2021 | Fatal |
| 9 | Guangxi | 08/17/2021 | Male | 55 | 08/17/2021 | Critical |
| 10 | Guangxi | 08/23/2021 | Male | 55 | 08/30/2021 | Severe |
| 11 | Guangxi | 08/25/2021 | Female | 48 | 08/29/2021 | Severe |
| 12 | Guizhou | 11/21/2020 | Male | 3 | 11/28/2020 | Fatal |
| 13 | Sichuan | 05/13/2021 | Male | 49 | 05/16/2021 | Fatal |
| 14 | Sichuan | 06/22/2021 | Male | 57 | 07/05/2021 | Critical |
| 15^b^ | Sichuan | 06/25/2021 | Female | 51 | 07/02/2021 | Fatal |
| 16^b^ | Sichuan | 06/30/2021 | Male | 55 | 07/04/2021 | Critical |
| 17 | Sichuan | 07/13/2021 | Female | 65 | 07/13/2021 | Critical |
| 18 | Hunan | 07/26/2021 | Female | 55 | 08/01/2021 | Severe |
| 19^b^ | Hunan | 08/02/2021 | Male | 54 | 08/02/2021 | Mild |
| 20^b^ | Hunan | 08/28/2021 | Female | 58 | Not available | Not available |
| 21^b^ | Hunan | 09/08/2021 | Female | 40 | 09/09/2021 | Severe |
| 22 | Hunan | 09/26/2021 | Male | 66 | 09/27/2021 | Severe |
| 23 | Hunan | 10/03/2021 | Female | 60 | 10/13/2021 | Critical |
| 24^b^ | Zhejiang | 12/15/2021 | Female | 51 | 12/18/2021 | Severe |

1. The information was obtained from the WHO and China CDC website.
2. Full genome sequences of the strains isolated from these cases are publicly available and are included in this study for comparison.
