# Supplementary figures and images for: Novel H5N6 reassortants bearing the clade 2.3.4.4b HA gene of H5N8 virus have been detected in poultry and caused multiple human infections in China
Source: Emerg Microbes Infect. 2022 Apr 25;11(1):1174–85. doi: 10.1080/22221751.2022.2063076 (PMC9126593; doi:10.1080/22221751.2022.2063076)

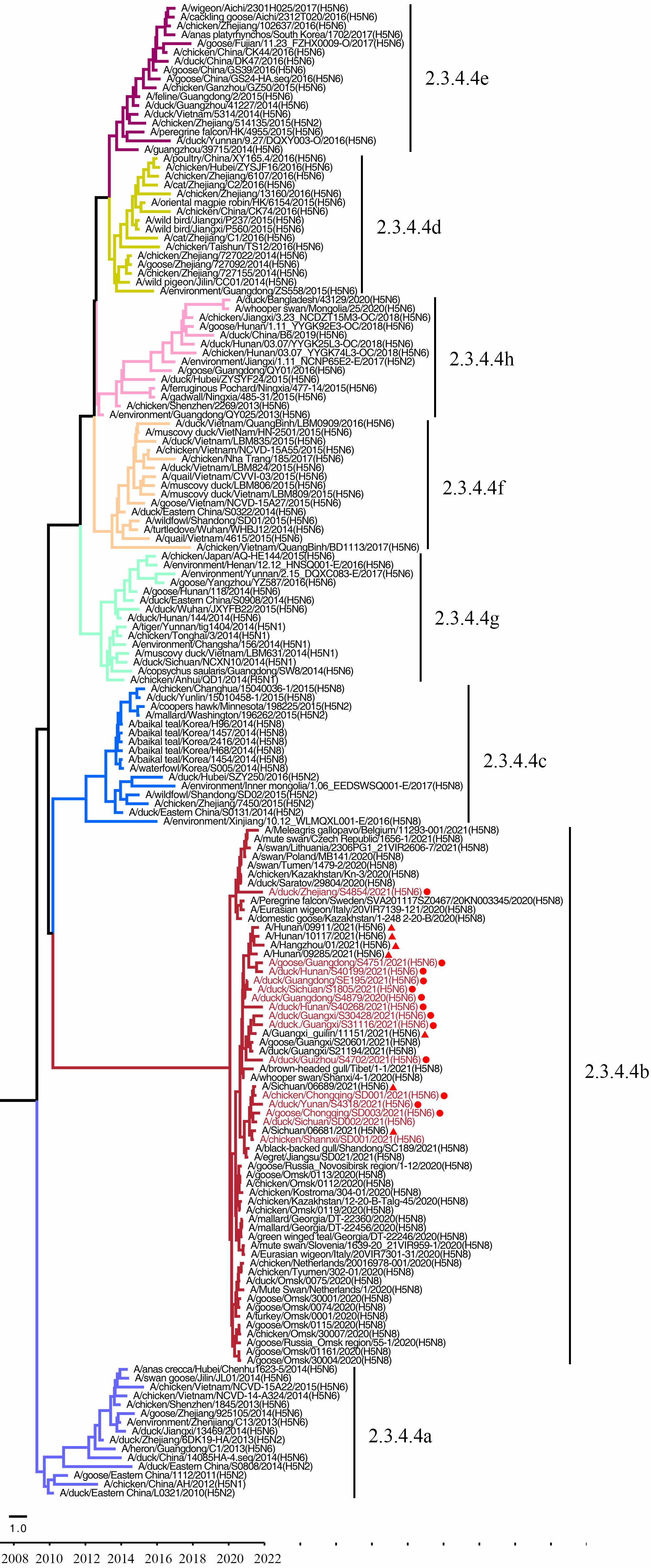

Supplement: Supplemental Material [file TEMI_A_2063076_SM3724.tif]
